# The Effect of Telephone Support Interventions on Coronary Artery Disease (CAD) Patient Outcomes during Cardiac Rehabilitation: A Systematic Review and Meta-Analysis

**DOI:** 10.1371/journal.pone.0096581

**Published:** 2014-05-05

**Authors:** Ahmed Kotb, Shuching Hsieh, George A. Wells

**Affiliations:** 1 Department of Epidemiology and Community Medicine, University of Ottawa, Ottawa, Canada; 2 Cardiovascular Research Methods Centre, University of Ottawa Heart Institute, Ottawa, Canada; University of Louisville, United States of America

## Abstract

**Background:**

Cardiac rehabilitation is offered to individuals after cardiac events to aid recovery and reduce the likelihood of further cardiac illness. However, patient participation remains suboptimal and the provision of high quality care to an expanding population of patients with chronic heart conditions is becoming increasingly difficult. A systematic review and meta-analysis was conducted to determine the effect of telephone support interventions compared with standard post-discharge care on coronary artery disease patient outcomes.

**Methods:**

The Cochrane Library, MEDLINE, EMBASE, and CINAHL were searched and randomized controlled trials that directly compared telephone interventions with standard post-discharge care in adults following a myocardial infarction or a revascularization procedure were included. Study selection, data extraction and quality assessment were completed independently by two reviewers. Where appropriate, outcome data were combined and analyzed using a random effects model. For each dichotomous outcome, odds ratios (OR) and 95% confidence intervals (CI) were derived for each outcome. For continuous outcomes, weighted mean differences (WMD) and standardized mean differences (SMD) and 95% CI were calculated.

**Results:**

26 studies met the inclusion criteria. No difference was observed in mortality between the telephone group and the group receiving standard care OR 1.12 (0.71, 1.77). The intervention was significantly associated with fewer hospitalizations than the comparison group OR 0.62 (0.40, 0.97). Significantly more participants in the telephone group stopped smoking OR 1.32 (1.07, 1.62); had lower systolic blood pressure WMD −0.22 (−0.40, −0.04); lower depression scores SMD −0.10 (−0.21, −0.00); and lower anxiety scores SMD −0.14 (−0.24, −0.04). However, no significant difference was observed for low-density lipoprotein levels WMD −0.10 (−0.23, 0.03).

**Conclusions:**

Compared to standard post-discharge care, regular telephone support interventions may help reduce feelings of anxiety and depression as well as, improve systolic blood pressure control and the likelihood of smoking cessation.

## Introduction

Cardiac rehabilitation (CR) is offered to individuals after cardiac events to aid recovery and reduce the likelihood of further cardiac illness. They have been previously shown to improve physical health as well as decrease subsequent morbidity and mortality through exercise, education, behavior change, counseling and other strategies aimed at targeting traditional risk factors for cardiovascular disease [1]–[6]. Despite these benefits however, patient participation in these programs remains suboptimal [7].

Some evidence suggests that interventions involving motivational communications delivered through letters, telephone calls and home visits may increase the uptake of cardiac rehabilitation [8].This offers promise as the provision of high quality care to an expanding population of older patients with chronic heart conditions becomes increasingly difficult. On the other hand, patients may be unwilling or unable to make frequent clinic attendance due to financial, transport or disability constraints [9].

To date, much of the evidence available has been focused on examining the effect of complex and multifactorial telemedicine interventions on heart failure (HF) patients. HF is a complex debilitating syndrome that results from a cardiac dysfunction that impairs the ability of the ventricle to fill with or eject blood[10]. More recently however, more basic telephone support interventions have been adapted for use in coronary artery disease (CAD) patient populations CAD is one of the most common forms of heart disease that results from an impedance or blockage of one or more arteries that supply blood to the heart [11]–[12].

Previous reports have examined the impact of multifaceted interventions on chronic diseases in general. When multifaceted interventions are examined, it becomes difficult to determine specifically which method of telemedicine appears most effective for this particular patient population. The aim of this systematic review and meta-analysis is to examine the literature on the impact of receiving structured telephone support, during cardiac rehabilitation, on clinical events, cardiac risk factors and patient reported outcomes in individuals with CAD compared to receiving usual follow-up care alone. The research questions addressed were: (1) What impact does structured telephone support (STS) have on mortality and hospitalization? (2) What impact does STS have on controlling risk factors such as smoking, systolic blood pressure, and low-density lipoprotein? (3) What does STS have on patient reported outcomes such as anxiety and depression?

## Methods

### Data Sources and Searches

Relevant randomized controlled trials published before September 2012 were identified by searching the following databases: Cochrane Central Register of Controlled Trials (CENTRAL), Database of Abstracts of Reviews of Effects (DARE) and Health Technology Assessment Database (HTA) on The Cochrane Library, MEDLINE, EMBASE, CINAHL, AMED, and the Web of Knowledge. Language restrictions were not applied to any of the searches. Bibliographies of included trials were examined to identify other potentially relevant studies.

### Study Selection

Randomized controlled trials were included if they directly compared the impact of telephone-delivered post-discharge interventions with standard care at discharge in adults (18 years or older) who had experienced a myocardial infarction (MI), a revascularization procedure (coronary artery bypass grafting (CABG) or percutaneous transluminal coronary angioplasty (PTCA)), and those with angina, or angiographically defined coronary heart disease. The primary outcome was all-cause hospitalization. Secondary outcomes included all-cause mortality, depression, anxiety as well as measures taken to reduce the risk of further cardiac illness such as smoking cessation, reducing systolic blood pressure, and low-density lipoprotein cholesterol levels.

In the first phase of screening, the titles and abstracts of all identified citations were screened by two independent reviewers (AK and SC). In the second phase of screening, full manuscripts were retrieved and screened by two independent reviewers on the basis of our predefined patient population, intervention, comparison, outcomes and study design of interest. Disagreements were resolved through discussion or through adjudication by a third reviewer (GW).

### Data Extraction and Quality Assessment

For each included paper, one review author (AK) extracted data and a second author (SC) checked the extracted data and disagreements were resolved by discussion between the two review authors. If no agreement could be reached, a third author (GW) was required for adjudication.

The SIGN-50 checklist and the Cochrane Collaboration's tool for assessing risk of bias (ROB) were used to evaluate the methodological quality of included trials. Two independent reviewers conducted the quality assessments (AK and SC). Disagreements between reviewers were resolved by discussion or through adjudication by a third reviewer (GW).

### Data Synthesis and Analysis

The primary analysis was a comparison of telephone follow-up with usual care. Heterogeneity amongst included studies was explored qualitatively by comparing the characteristics of included studies, visual inspection of forest plots and quantitatively using Cochrane's Q test and I^2^ statistic. For continuous data (using the same measuring instrument) the weighted mean difference (WMD) and 95% confidence intervals (CI) are reported. Where the studies have used different instruments to measure the same conceptual outcome, the standardized mean difference (SMD) is reported. In studies that report dichotomous data, the odds ratios (OR) or risk ratios (RR) and confidence intervals (CI) are reported. To account for heterogeneity and take a more conservative approach, the analyses were carried out using the random-effects model are presented. Sensitivity analyses using fixed effect models were conducted for comparison.

## Results

### Search results

**Figure 1:**
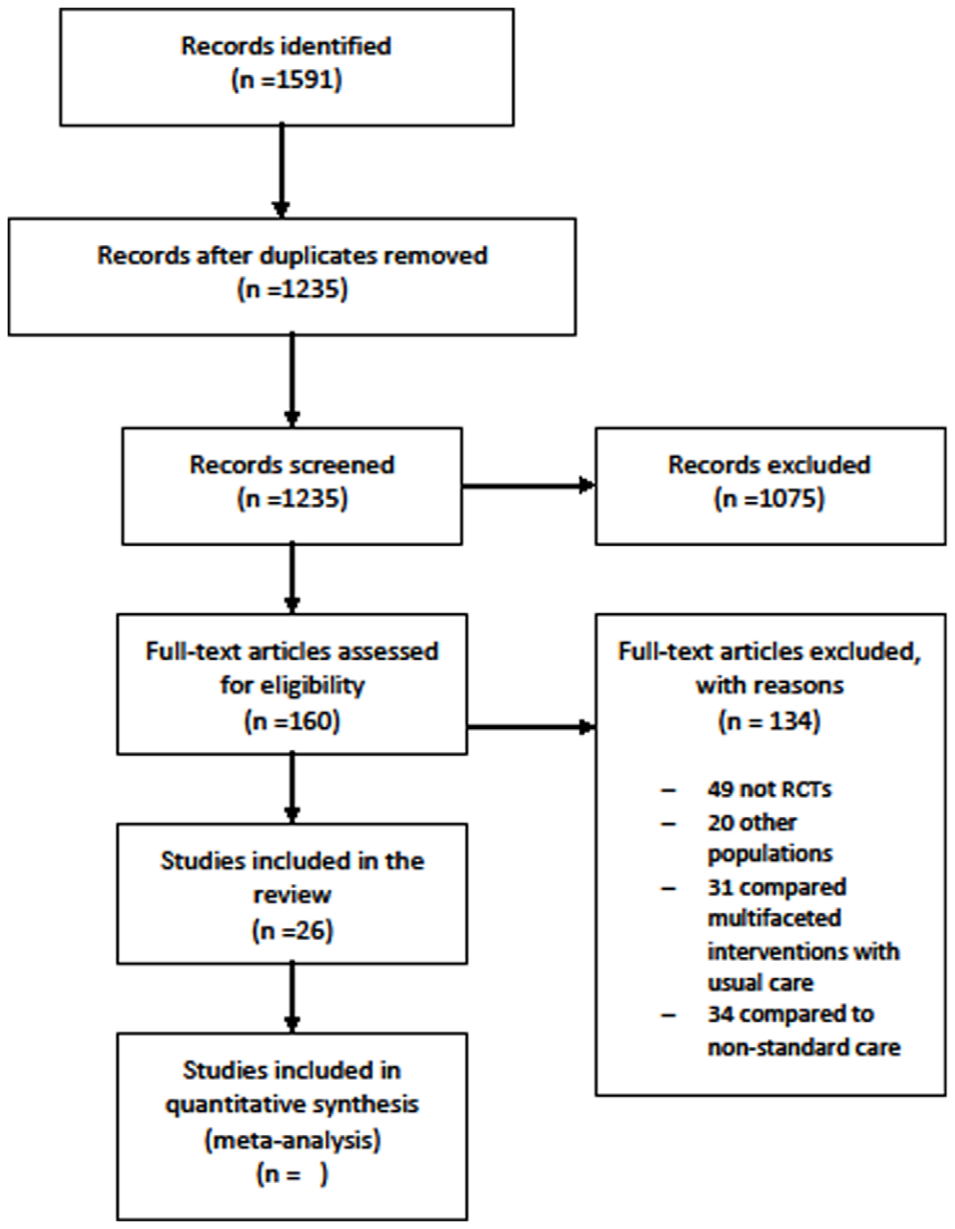
Modified PRISMA diagram.

The electronic search conducted yielded a total of 1,538 titles. The reference lists of studies later included were hand-searched and resulted in the selection of 53 studies for additional screening. After duplicates were removed, the titles and abstracts of 1,235 studies were screened. A total of 1,075 studies were excluded and 160 studies were retrieved for possible inclusion. After examining their full texts, 26 studies were included [13]–[38] and 134 were excluded. The study selection process and the reasons for exclusion are summarized in the PRISMA flow diagram shown in Figure 1.

### Description of studies

All included randomized controlled trials (4,081 participants) compared a telephone intervention designed to improve cardiac patients' outcomes directly to standard post-discharge care. Nine of the included studies were conducted in Canada [13], [16], [20], [30], [31], [32], [33], [34], [35] 8 in Australia[17], [25]–[29], [37], [38], 5 in the United States of America[14], [21]–[23], [38] 3 in Europe [18], [19], [24] and 1 in Iran [15]. Thirteen studies had longer than 6 months of follow-up [19], [21], [22], [24], [25], [26], [28], [29], [30], [31]–[33], [38]. Seven studies reported less than 6 months of follow-up [15]–[17], [20], [27], [34], [35] and 6 reported outcomes at 6 months [13], [14], [18], [23], [36], [37]. Sample sizes varied considerably across studies (range: 59 to 792) as well as the number of calls made to participants (range: 3 to 24).

Of the 26 included studies, 8 studies recruited patients diagnosed with Acute Coronary Syndrome [14], [21], [23], [25]–[29] 8 recruited patients who had undergone revascularization patients [13], [15], [16], [20], [31]–[33], [37] 4 studies recruited patients diagnosed with a myocardial infarction [18], [19], [34], [38] and 6 recruited any patients diagnosed with coronary artery disease [17], [22], [24], [30], [35], [36]. Ten studies described their patient populations as having received some degree of cardiac rehabilitation [13], [15], [17], [19], [24], [31]–[33], [36], [37]. Of those 10 studies, only 7 provided the proportion of patients who participated in CR (range: from 32% to 100%) [17], [24], [31]–[33], [36], [37]. Five studies described their patient populations as not accessing CR [25]–[29] and the remaining 11 studies did not provide detail regarding how much of their included participants also took part in cardiac rehabilitation [14], [16], [18], [20]–[23], [30], [34], [35], [38].

The frequency of calls made varied between 3–6 times in fourteen studies and was greater than 6 calls in five studies. In 8 studies, the frequency of the intervention was not detailed [18], [19], [22], [28], [34], [35], [36], [38]. In most studies, the telephone support intervention was delivered by a clinician with nurses being the most commonly reported delivery personnel. The second most common professional delivering the intervention was an exercise specialist. This occurred when the interventions' main component was exercise [13], [31]–[33]. In one instance, when the intervention was focused primarily on lowering cholesterol levels, the intervention was delivered by a dietitian [37]. In one instance when the intervention was designed to address a multitude of risk factors, the intervention was delivered by a health educator. Further detail is available in Table 1 regarding the design of each included study, the type of patients included, and the interventions compared.

**Table 1:** Characteristics of included studies.

Author/Year	Country	Population	Comparisons	Follow-up	Quality
1.Arthur 2002 ^13^	Canada	CABG patients (N = 242). Participating in a Cardiac Rehabilitation program: Yes	Intervention: In addition to exercise, patients were telephoned every 2 weeks by the exercise specialist. Comparison: Hospital based exercise training	6 months	High quality
2.Bambauer 2005 ^14^	USA	ACS patients (N = 100). Participating in a Cardiac Rehabilitation program: Not described	Intervention: Six 30 minute telephone counseling sessions. Comparison: Patients received a booklet on coping with chronic illness and were instructed to contact their primary care physician if they experienced any warning signs of more significant depression.	6 months	Acceptable
3.Bazargani 2011 ^15^	Iran	CABG patients (N = 300). Participating in a Cardiac Rehabilitation program: Yes	Intervention: 6 sessions (150 min/week) of psycho-education. Comparison: Not described	3 months	Unacceptable
4.Beckie 1989 ^16^	Canada	CABG patients (N = 74). Participating in a Cardiac Rehabilitation program: Not described	Intervention: 4 to 6 supportive-educative telephone calls with a cardiac rehabilitation nurse specialist. Comparison: Received routine in-hospital teaching available to all patients undergoing cardiac surgery.	1.5 months	Acceptable
5.Gallagher 2003 ^17^	Australia	Women with CAD (N = 196). Participating in a Cardiac Rehabilitation program: 32% did	Intervention: 4 telephone calls to assist coping with recovery. Comparison: All inpatients received a Phase I education program, and all women were referred to local cardiac rehabilitation programs.	3 months	Acceptable
6.Hanssen 2007 ^18^	Norway	AMI patients (N = 288). Participating in a Cardiac Rehabilitation program: Not described	Intervention: Nurse-initiated telephone calls after discharge. Comparison: All patients in the control group were managed in accordance with the current clinical practice, which encompassed one visit to a physician at the outpatient clinic 6–8 weeks after discharge, and subsequent visits to the patient's general practitioner.	6 months	Acceptable
7.Hanssen 2009 ^19^	Norway	AMI patients (N = 288). Participating in a Cardiac Rehabilitation program: A very small proportion were referred	Intervention: Nurse-initiated telephone calls after discharge. Comparison: All patients in the control group were managed in accordance with the current clinical practice, which encompassed one visit to a physician at the outpatient clinic 6–8 weeks after discharge, and subsequent visits to the patient's general practitioner.	18 months	Acceptable
8.Hartford 2002 ^20^	Canada	CABG patients (N = 166) who have a caregiver. Participating in a Cardiac Rehabilitation program: Not described	Intervention: 6 telephone calls to patients and partners. Comparison: The control group received usual care, which did not include systematic follow-up	2 months	High quality
9.Holmes-Rovner 2008 ^21^	USA	ACS patients (N = 525). Participating in a Cardiac Rehabilitation program: Not described	Intervention: Six-session telephone counseling calls by a health educator. Comparison: Patients received a written discharge contract listing recommended outpatient medications, cardiac rehabilitation recommendations, and health behavior changes (smoking cessation, diet modification, and exercise), as well as numerical values for ejection fraction and cholesterol.	8 months	Acceptable
10.Ma 2010 ^22^	USA	CAD patients (N = 689). Participating in a Cardiac Rehabilitation program: Not described	Intervention: Pharmacist-delivered telephone counseling calls. Comparison: consisted of normal clinical care as determined by the patient's provider.	12 months	Acceptable
11.Mclaughlin 2005 ^23^	USA	ACS patients (N = 100) with symptoms of depressive illness or anxiety. Participating in a Cardiac Rehabilitation program: Not described	Intervention: 3–6 telephone counseling sessions of 30 minutes by clinicians. Comparison: Patients received a booklet on coping with cardiac illness typical of those given at hospital discharge and were instructed to contact their primary care physician if they experienced any warning signs of depression.	6 months	Acceptable
12.Mittag 2006 ^24^	Germany	CAD patients (N = 343). Participating in a Cardiac Rehabilitation program: All received 3 weeks of inpatient Cardiac Rehabilitation	Intervention: Monthly nurse-initiated telephone contacts. Comparison: The control group received six flyers on general health topics (relaxation, sports and physical exercise, sleep disorders, low back pain, nutrition) by mail every second month as an attention placebo. Patients in the intervention group were given the same written information	12 months	Acceptable
13.Neubeck 2009 ^25^	Australia	ACS patients (N = 208). Participating in a Cardiac Rehabilitation program: Not accessing CR	Intervention: A clinic visit plus 3 months of phone support. Comparison: ongoing conventional health care. Managing cardiovascular health in consultation with their GP and cardiologist.	48 months	Acceptable
14.Neubeck 2011 ^26^	Australia	ACS patients (N = 208). Participating in a Cardiac Rehabilitation program: Not accessing CR	Intervention: 1-hour consultation and telephone calls over 3 months. Comparison: ongoing conventional health care. Managing cardiovascular health in consultation with their GP and cardiologist.	48 months	High quality
15.Redfern 2008 ^27^	Australia	ACS patients (N = 208). Participating in a Cardiac Rehabilitation program: Not accessing CR	Intervention: 1-hour consultation and approximately four 10-minute follow-up calls. Comparison: Participants continued to manage their cardiovascular health as directed by their family physician often in consultation with their cardiologist.	3 months	High quality
16.Redfern 2009 ^28^	Australia	ACS patients (N = 208). Participating in a Cardiac Rehabilitation program: Not accessing CR	Intervention: Clinic visit plus telephone support and tailored preferential risk modification. Comparison: continuing conventional care but no centrally coordinated secondary prevention	12 months	High quality
17.Redfern 2010 ^29^	Australia	ACS patients (N = 208). Participating in a Cardiac Rehabilitation program: Not accessing CR	Intervention: One-hour initial consultation and four 10 minute follow-up phone calls over three months. Comparison: participated in ongoing conventional care, aimed at managing their cardiovascular health as directed by their General Practitioner, ideally in consultation with their Cardiologist.	12 months	High quality
18.Reid 2007 ^30^	Canada	CAD patients (N = 100) who were also current smokers. Participating in a Cardiac Rehabilitation program: Not described	Intervention: Automatic telephone contact plus counseling by up to three 20-min telephone sessions. Comparison: All participants received advice to quit smoking; access to Nicotine Replacement Therapy during hospitalization (if necessary); brief bedside counseling with a nurse-specialist; a self-help guide; and the provision of information about the hospital's outpatient smoking cessation program and other community programs.	12 months	High quality
19.Smith 2004 ^31^	Canada	CABG patients (N = 222). Participating in a Cardiac Rehabilitation program: All participated in CR (home vs. hospital-based)	Intervention: Exercise program and telephone follow-up every 2 weeks by an exercise specialist. Comparison: Patients assigned to the Hospital based exercise group were expected to attend supervised exercise sessions 3 times per week for 6 months.	12 months	High quality
20.Smith 2007 ^32^	Canada	CABG patients (N = 196). Participating in a Cardiac Rehabilitation program: All participated in CR (home vs. hospital-based)	Intervention: Exercise program and telephone follow-up every 2 weeks by an exercise specialist. Comparison: Patients assigned to the Hospital based exercise group were expected to attend supervised exercise sessions 3 times per week for 6 months.	72 months	High quality
21.Smith 2011 ^33^	Canada	CABG patients (N = 196). Participating in a Cardiac Rehabilitation program: All participated in CR (home vs. hospital-based)	Intervention: Exercise program and telephone follow-up every 2 weeks by an exercise specialist. Comparison: Patients assigned to the Hospital based exercise group were expected to attend supervised exercise sessions 3 times per week for 6 months.	72 months	High quality
22.Stevens 1985 ^34^	Canada	MI patients (N = 59). Participating in a Cardiac Rehabilitation program: Not described	Intervention: Received telephone calls by 2 nurses and the investigator. Comparison: nurses educated MI patients prior to discharge and all got a booklet to take home. Upon discharge patients were returned to the care of the GP and received usual follow-up.	1.5–2 months	High quality
23.Tranmer 2004 ^35^	Canada	CAD patients (N = 200). Participating in a Cardiac Rehabilitation program: Not described	Intervention: Follow-up via nurse-initiated telephone calls. Comparison: Usual care included preoperative and discharge preparation by the nurse, provision of an education booklet and home care follow-up, as necessary.	1.25 months	High quality
24.Vale 2003 ^36^	Australia	CAD patients (N = 792). Participating in a Cardiac Rehabilitation program: 53% of patients in the intervention group and 57% of the patients in the control group attended a cardiac rehabilitation program.	Intervention: Patients received coaching sessions by telephone. Comparison: Patients received a hospital discharge summary, a one page chart of risk factor for CHD secondary prevention to them and their medical caregivers as well as contacted once after discharge at 24 weeks for follow-up assessment	6 months	High quality
25.Vale 2002 ^37^	Australia	CABG or PCI patients (N = 245). Participating in a Cardiac Rehabilitation program: 53% of patients in the intervention group and 50% of the patients in the control group attended a cardiac rehabilitation program.	Intervention: Dietitian contacted patients 5 times by telephone regarding lipid levels. Comparison: All patients in the study (including patients in the coaching intervention group) were offered information about a cardiac rehabilitation program and were encouraged to attend. Patients in the usual care group were contacted at 24 weeks postrandomization to obtain a fasting serum lipid profile within the next 2 weeks.	6 months	High quality
26.Van Elderen 1994 ^38^	USA	AMI patients (N = 60). Participating in a Cardiac Rehabilitation program: Not described	Intervention: Nurse contacted the patient by telephone. Comparison: Patients received standard medical care only; consisting primarily of medical care. A standard physical rehabilitation program was mplemented in the nursing ward.	12 months	Acceptable

**Note:** Studies underlined and in bold were included in the meta-analysis. The other studies were described qualitatively. CABG = Coronary artery bypass graft. ACS = Acute coronary syndrome. AMI = Acute myocardial infarction. CAD = Coronary artery disease. PCI = Percutaneous coronary intervention.

### Risk of bias in included studies

**Figure 2:**
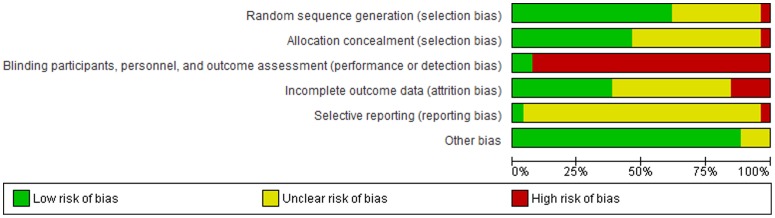
Risk of bias graph.

Using the SIGN-50 quality assessment tool for randomized controlled trials, 14 studies were considered to be of high quality [13], [20], [26]–[37] 11 were considered to be of acceptable quality[14], [16]–[19], [21]–[25], [38] and 1 was considered to be low [15] (see Table 1). A summary of the risk of bias of included studies is described in Figure 2. The risk of bias assessment of each study is detailed in Figure S1. Funnel plots were only considered for the outcome of mortality due to the fact that the number of studies was deemed sufficient to produce a reliable assessment (Figure S2). No considerable asymmetry was apparent.

#### Structured telephone support interventions versus usual care

**Figure 3:**
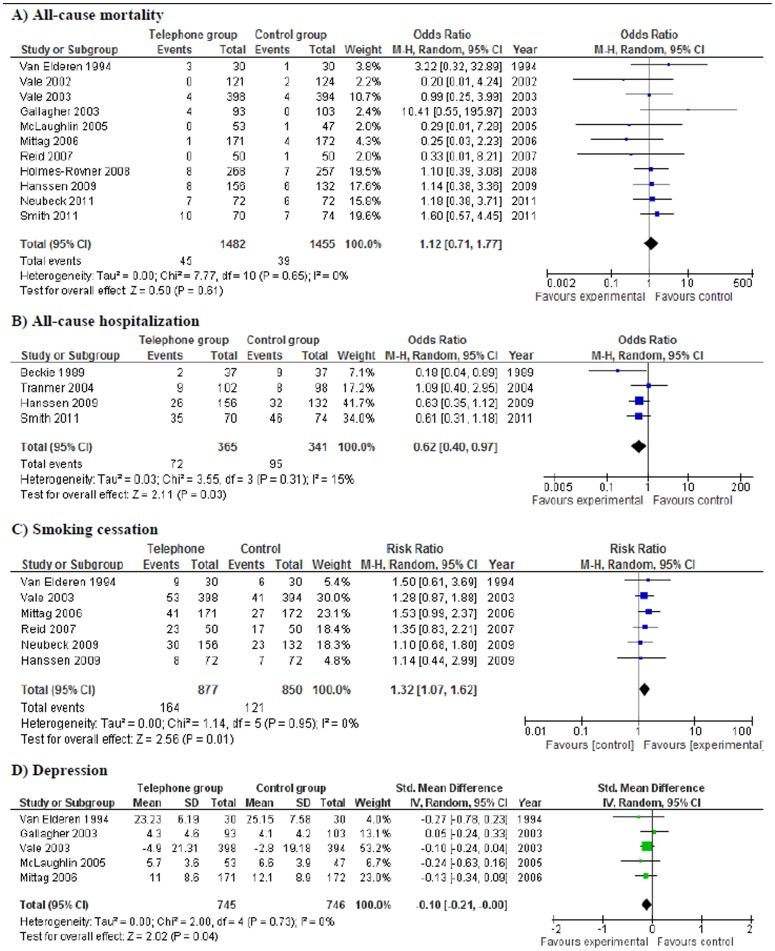
Comparing telephone support with usual care.

Figure 3 provides a summary of the intervention's main effects compared to usual care for the following outcomes of interest: all-cause mortality, all-cause hospitalization, smoking cessation, and depression. Further detail on the meta-analysis of the following outcomes: systolic blood pressure, low-density lipoprotein levels, and anxiety are available in Figures S3-S8.

### Clinical events

Data on all-cause mortality was available and considered appropriate to be combined across 11 studies (Figure 3). Five studies were conducted in N. America, 4 in Australia and 2 in Europe. The quality was judged to be high for 5 studies and acceptable for 6. With the exception of one study, all included studies followed patients for at least 6 months. Four studies described their included patient population as having had an acute myocardial infarction, 4 as having acute coronary syndrome and 3 as having had a revascularization procedure. In 5 studies, a significant proportion of the included population participated in a cardiac rehabilitation program. In the remaining six studies, the participation of patients in a cardiac rehabilitation program was not described. In 7 of the studies the intervention was delivered by a nurse who offered support and education on topics that included risk factor control and improved symptom recognition. In the remaining 4 studies, the professionals delivering the intervention included health educators, pharmacists, dietitians, and exercise specialist. When the intervention was delivered by an exercise specialist the intervention focused more on physical activity in the period following an event. Where the intervention was delivered by a dietitian or pharmacist the focus shifted more towards lipid control. The I^2^  =  0% and the overall effect estimate found showed no difference in the odds of mortality between the intervention and comparison group [OR 1.12 95% CI (0.71, 1.77)].

A total of 4 studies reported on hospitalization after discharge (Figure 3). Three of the four studies were conducted in Canada and one was conducted in Norway. Three of the four studies were considered to be of high quality and the fourth study was judged to be of acceptable quality. Heterogeneity was further examined according to the PICO statement of individual trials. In three out of the four studies, the majority of the CAD patient population had undergone a revascularization procedure. With the exception of the study by Smith (2011), three out of the four studies involved telephone follow-up carried out by a nurse that focused on the provision of support and education. Furthermore, only the study by Smith (2011) included a large portion of individuals who were participating in a cardiac rehabilitation program. The statistical measure of heterogeneity was low (I^2^ = 15%) and the overall effect estimate indicated significantly lower odds of hospitalization [OR 0.62 95% CI (0.40, 0.97)] in the telephone group. It is important to note however, that when a sensitivity analysis was conducted with and without the most outlying study, Beckie (1989), the significant effect was no longer found [OR 0.68 95% CI (0.45, 1.01)].

#### Modifiable risk factors

A total of 6 studies reported data on smoking cessation (Figure 3). Two studies were conducted in N. America, 2 in Europe and 2 in Australia. All six studies were considered to be of either acceptable or high quality. The follow-up period in all six studies was 6 or more months. In all six studies, the patient population was described as individuals recovering from either an AMI or CABG procedure. In all studies, the intervention was delivered by nurses who took part in coaching, supporting and educating participants. In only two studies, participants were described as having received cardiac rehabilitation. When combined together the overall effect estimate indicated significantly greater odds of smoking cessation in the group receiving the telephone intervention [Risk Ratio 1.32 95% CI (1.07, 1.62)].

Two studies reported data on SBP differences between treatment groups. The follow-up period in both studies was 12 or more months. The study by Mittag (2006) was conducted in Germany and the study by Neubeck (2011) was conducted in Australia. The quality of both studies was considered to be acceptable. Both studies included acute CAD patients and both telephone follow-up interventions were delivered by nurses and focused on risk factor reduction. The I^2^  =  0% and the overall calculated WMD for SBP was significantly lower for the telephone group [WMD −4.22 95% CI (−7.58, −0.85)].

A total of 4 studies reported data regarding the change in LDL levels between treatment groups. In three out of the 4 studies the follow-up period was greater than or equal to 12 months. Two studies were conducted in N. America and two were conducted in Australia. The quality of the studies was considered high in 3 studies and acceptable in 1. Three studies described their patient population as acute coronary syndrome or recovering from a revascularization procedure while one study only broadly defined patients as having coronary heart disease (CHD). The telephone intervention was delivered by a different type of specialist in each study. This included a dietitian, an exercise specialist, a nurse, and a pharmacist. The I^2^ statistic  =  71% when these studies were analyzed together using a random effects model. When only studies of longer than 6 months follow-up were examined, the I^2^ statistic was reduced to 16% and the overall WMD for LDL was not found to significantly differ between comparison groups (WMD −0.07 [−0.20, 0.05]).

#### Patient reported outcomes

In total 5 studies measured and reported on the outcome of depression (Figure 3). In 4 out of the 5 studies, the follow-up period was greater than or equal to 6 months. Two studies were conducted in the United States, two in Australia and one in Germany. The quality of the studies was judged to be acceptable in 4 studies and high in one. In three studies, the patient populations were described to have an acute myocardial infarction (AMI). The patient populations in the remaining two studies were described as having an acute coronary syndrome (ACS) and having undergone was revascularization. In 3 out of 5 studies, a significant portion of the patients received some cardiac rehabilitation services. In 4 out of 5 studies the intervention was delivered by a nurse. In the study that did not involve nurses in the delivery, the intervention was delivered by clinicians. The I^2^ statistic  =  0% and the overall calculated SMD showed a significantly lower (p  =  0.04) depression score in the telephone group than the comparison [SMD −0.10 95% CI (−0.21, −0.00)].

Six studies examined the impact of regular telephone follow-up on feelings of anxiety. In 4 out of the 6 studies the follow-up period was greater than or equal to 6 months. Three studies were conducted in N. America, 2 in Australia and 1 in Europe. The quality of the studies was judged to be acceptable in 5 studies and high in one. The patient population was described as having had an AMI or CABG in 5 studies and as ACS in one. In 3 out of 6 studies, a significant portion of the included population received some cardiac rehabilitation services. In 5 out of 6 studies, the nurses delivered the telephone support intervention and in one study the intervention was delivered by a clinician.

Even though the overall calculated SMD indicated that participants in the telephone group had significantly lower anxiety scores than those in the comparison group [SMD −0.29 95% CI (−0.56, −0.01)], the forest plot and I^2^ (I^2^  =  81%) indicated that a considerable amount of heterogeneity was evident across studies. The most outlying study by Beckie (1989) had the shortest follow-up having only followed patients for a period of 6 weeks and included CAD patients who were less severe or acutely ill than the patients in other studies. This study was excluded from subsequent analyses that examined studies of longer than 6 weeks of follow-up.

When studies of at least 3 months of follow-up were examined, the analysis included 5 out of the 6 studies. The I^2^  =  29% and the telephone group was found to have reduced feelings of anxiety than the control group [SMD −0.14 95% CI (−0.24, −0.04)]. This effect remained when studies of 6 or more months of follow-up were examined. This analysis included 4 out of 6 studies and demonstrated that the participants in the telephone intervention group had significantly lower anxiety scores [SMD −0.18 95% CI (−0.30, −0.07)].

## Discussion

Many patients with CAD continue to face challenges maintaining their adherence to recommendations for risk reduction such as managing their blood pressure, lowering their low-density lipoprotein levels and abstaining from smoking. A wealth of available evidence also suggests a strong link between increased feelings of depression and anxiety in the period that follows having a coronary event. Together, these continued challenges place individuals with these diseases at an increased risk of further cardiac illness and death. The hypothesis in this review was that the availability of remote monitoring and support services for recovering patients may facilitate access to care and improve patients' outcomes through cardiac risk reduction and improved patient outcomes.

Study participants were mostly males, aged between of 50 and 70 years old, and diagnosed as acute CAD patients defined as having had an MI or a revascularization procedure. With the exception of one study that was conducted in the Middle East, all studies were conducted in either N. America, Europe or Australia. With the exception of one study, the quality of included studies was either high or acceptable and the follow-up period was typically six or more months. The telephone support intervention was typically delivered by a nurse who supported patients and educated them on matters that included cardiac risk reduction and improved symptom recognition. Outcomes considered included clinical events (all-cause mortality and hospitalization), modifiable risk factors (smoking cessation, low-density lipoprotein, and systolic blood pressure), and other patient outcomes (depression and anxiety).

No evidence was found to support any additional benefit as a result of the telephone intervention in terms of a reduction in mortality and level of low-density lipoprotein. These findings were consistent with findings by Neubeck (2009) and Whalley (2011) that showed no strong evidence for reductions in total deaths and the review by Taylor (2010) that found no difference between groups in terms of LDL levels [40], [41].

In this review, participants receiving the telephone intervention did however have significantly fewer hospitalizations. They also experienced significant reductions in systolic blood pressure and were more likely to stop smoking. These findings were similar to those by Barth (2008) and by Neubeck (2009), where telephone support was found to significantly promote smoking cessation in patients with coronary heart disease [11], [39]. Neubeck (2009) also demonstrated that participants in the telephone group had significantly lower systolic blood pressure.

Patients receiving the telephone intervention also had significantly lower depression and anxiety scores were observed in participants who received the telephone intervention. Symptoms of anxiety and depression are commonly experienced by patients with coronary artery diseases (CAD). Depression and anxiety have been previously associated the with increased severity of CAD, the number and length of cardiac-related hospitalizations and all-cause mortality, and can predict greater risk major adverse cardiac events in patients with stable CAD[42]–[44]. Evidence from this systematic review and meta-analysis is therefore in support of conducting a randomized controlled trial of sufficient power and at least 12 months of follow-up to compare the impact associated with the delivery of a regular telephone intervention alongside usual care for monitoring and supporting coronary artery disease patients following an acute cardiac event or revascularization procedure.

This review has several important limitations to consider. Like any systematic review, the strengths of the results depends primarily on the quality and completeness of the data currently available from included studies. Detailed descriptions around participants' attendance and compliance rates for cardiac rehabilitation programs were inadequately reported. This limited how thoroughly this issue can investigated in order to determine if the benefits associated with telephone support are perhaps due to an increased participation in cardiac rehabilitation programs. Furthermore, intensive monitoring can add to more contact with providers and in some instances, more tests. This can occur for an intervention of this sort. Almost all studies were conducted in what are considered to be high-income countries. This in turn limits the generalizability of the findings to settings outside of N. America, Europe and Australia. As is commonly expected, the patient populations varied slightly across included studies. Although only patients with an acute form of coronary artery disease patients who had not advanced to heart failure were considered for this review, included studies described their patients as having either had an acute myocardial infarction, diagnosis of acute coronary syndrome or had undergone a revascularization procedure. A randomized controlled trial that compares the effect of standard care and a telephone support intervention delivered by nurses to aid in the education and counseling of either one of the aforementioned patient groups therefore remains warranted. Even though such resource utilization implications may compromise the benefits associated with telephone support, there was no data available from included studies to address this concern. Further research is needed to examine the cost-effectiveness of this intervention as it compares to the current standard of post-discharge care.

## Conclusions

The effectiveness of this simple telephone intervention is of relevance given that most cardiac rehabilitation programs involve one or more of the following: routine monitoring, counseling, and educating. Some of these benefits can be feasibly delivered remotely using telephone technology as a medium. Evidence from this review suggests that telephone support and monitoring appear more effective in reducing certain risk factors than others, physicians may identify, depending on each patient's rehabilitation goals, which patients would be most likely to benefit from the intervention. Through reducing feelings of anxiety and depression, improved control over cardiac risk reduction and fewer hospitalizations, structured telephone support and follow-up can aid in the delivery of specialist preventive care to patients who may otherwise not have access to them and may have the potential to reduce some of the burden on the healthcare system. If hospitalization, anxiety and depression are indeed reduced this would be extremely valuable and possibly cost effective. A larger definitive randomized controlled trial of this intervention targeted to a specific population likely to benefit most is therefore merited.

## Supporting Information

Checklist S1 **PRISMA Checklist.**
(DOCX)

Figure S1 **Risk of bias summary.**
(TIF)

Figure S2 **Funnel plot.**
(TIF)

Figure S3 **Comparison of structured telephone support and usual care on systolic blood pressure.**
(TIF)

Figure S4 **Comparison of structured telephone support and usual care on low-density lipoprotein.**
(TIF)

Figure S5 **Sensitivity analysis comparing structured telephone support and usual care on LDL levels in studies of at least 6 months of follow-up.**
(TIF)

Figure S6 **Comparison of structured telephone support and usual care on anxiety.**
(TIF)

Figure S7 **Sensitivity analysis comparing structured telephone support and usual care on anxiety in studies of at least 3 months of follow-up.**
(TIF)

Figure S8 **Sensitivity analysis comparing structured telephone support and usual care on anxiety in studies of at least 6 months of follow-up.**
(TIF)
