# Supplementary figures and images for: The Effect of Telephone Support Interventions on Coronary Artery Disease (CAD) Patient Outcomes during Cardiac Rehabilitation: A Systematic Review and Meta-Analysis
Source: PLoS One. 2014 May 5;9(5):e96581. doi: 10.1371/journal.pone.0096581 (PMC4010507; doi:10.1371/journal.pone.0096581)

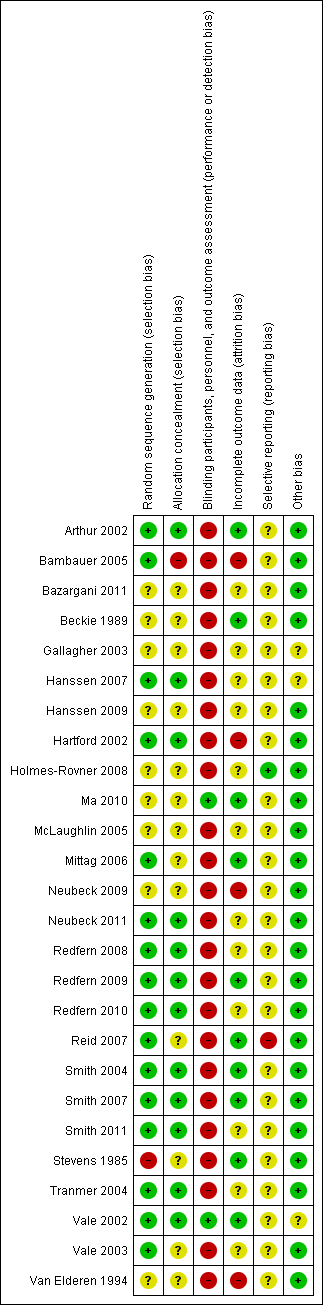

Supplement: Figure S1 — Risk of bias summary. (TIF) [file pone.0096581.s002.tif]

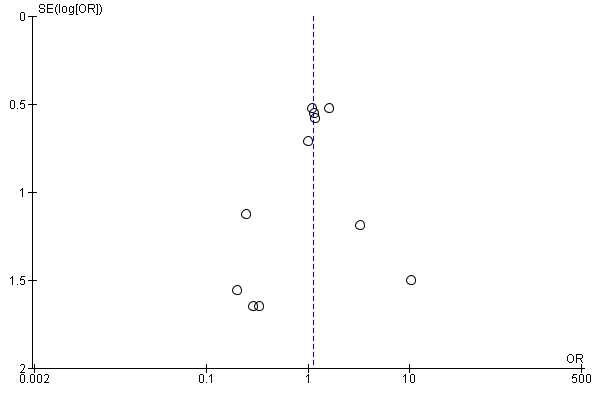

Supplement: Figure S2 — Funnel plot. (TIF) [file pone.0096581.s003.tif]

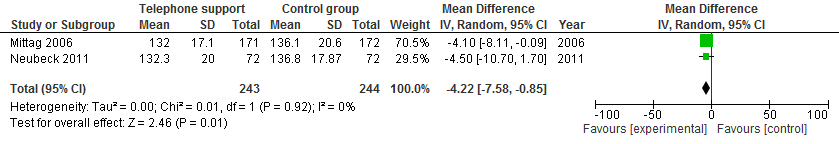

Supplement: Figure S3 — Comparison of structured telephone support and usual care on systolic blood pressure. (TIF) [file pone.0096581.s004.tif]

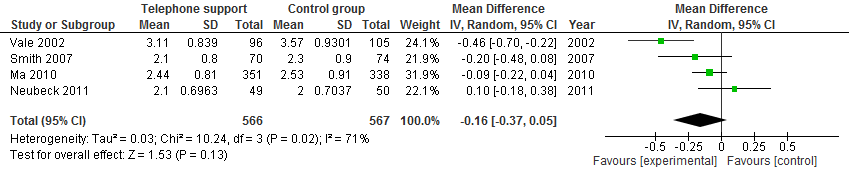

Supplement: Figure S4 — Comparison of structured telephone support and usual care on low-density lipoprotein. (TIF) [file pone.0096581.s005.tif]

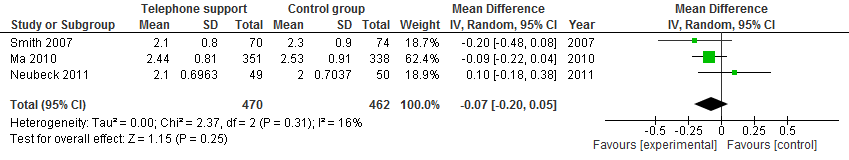

Supplement: Figure S5 — Sensitivity analysis comparing structured telephone support and usual care on LDL levels in studies of at least 6 months of follow-up. (TIF) [file pone.0096581.s006.tif]

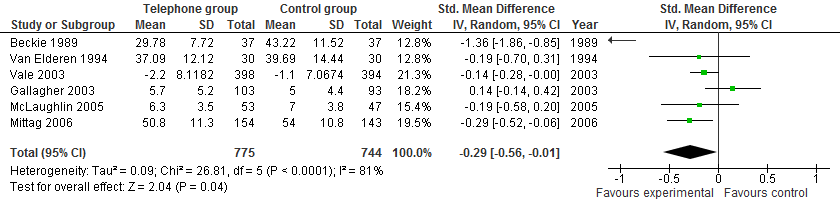

Supplement: Figure S6 — Comparison of structured telephone support and usual care on anxiety. (TIF) [file pone.0096581.s007.tif]

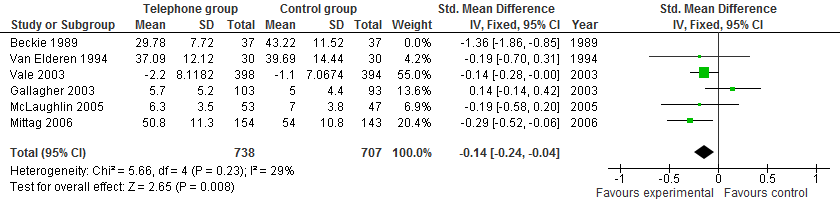

Supplement: Figure S7 — Sensitivity analysis comparing structured telephone support and usual care on anxiety in studies of at least 3 months of follow-up. (TIF) [file pone.0096581.s008.tif]

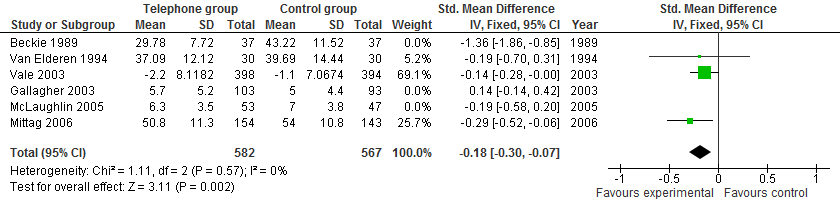

Supplement: Figure S8 — Sensitivity analysis comparing structured telephone support and usual care on anxiety in studies of at least 6 months of follow-up. (TIF) [file pone.0096581.s009.tif]
